# Diet quality measured using the Mediterranean diet score and anxiety during armed conflict

**DOI:** 10.3389/fnut.2026.1802921

**Published:** 2026-07-28

**Authors:** Mona Boaz, Daniela Abigail Navarro, Vered Kaufman-Shriqui

**Affiliations:** Department of Nutrition Sciences, Faculty of Health Sciences, Ariel University, Ariel, Israel

**Keywords:** anxiety, armed conflict, diet quality, Mediterranean diet, public health

## Abstract

**Background:**

Processed diets, particularly those high in fat, added sugars, and refined carbohydrates, are associated with increased anxiety, whereas dietary patterns rich in vegetables, fruits, dairy, nuts, and seeds appear protective. Mediterranean-style diets, characterized by high consumption of plant foods and olive oil with moderate intake of fish and poultry, are associated with reduced cardiometabolic, oncologic, and neurocognitive risks. Prior studies have linked adherence to Mediterranean-style diets with reduced anxiety; however, it remains unclear whether this association persists during periods of severe external stress, such as armed conflict.

**Objectives:**

To evaluate whether diet quality, assessed via adherence to a Mediterranean-style diet, was associated with anxiety levels among Israeli adults during the Iron Swords War (2023–2025).

**Methods:**

This cross-sectional online survey was conducted in a representative stratified (age, sex, ethnicity) random sample of Israeli adults from November 6–14, 2023. Participants completed validated measures assessing anxiety [General Anxiety Disorder (GAD-7)], diet quality [Israel Mediterranean diet screener (I-MEDAS)], food insecurity (Hunger Vital Sign), and demographic and lifestyle characteristics. Statistical analyses included *t*-tests, Mann–Whitney *U* tests, chi-square tests, and logistic regression.

**Results:**

Among 534 respondents, 39.7% exhibited moderate to severe anxiety (GAD-7 ≥ 10). Total I-MEDAS scores did not differ by anxiety level; however, poorer diet quality indicators—including reduced fruit and vegetable intake, increased snacking, self-reported dietary deterioration and weight gain—were significantly more common among highly anxious participants. Food insecurity, weight gain, younger age, and female sex independently predicted moderate to severe anxiety.

**Conclusion:**

While overall Mediterranean diet adherence was not associated with anxiety level, multiple indicators of diet deterioration were strongly linked to higher anxiety during armed conflict. These findings support the importance of public health strategies targeting both nutrition and mental health during periods of societal stress.

## 1 Introduction

Diets typified by elevated consumption of fat, added sugars and refined carbohydrates are associated with increased anxiety risk (1). Conversely, diets high in vegetables, fruits, milk, nuts, and seeds are associated with a lower risk of incident anxiety (2). Mediterranean-style diets are characterized by several daily servings of vegetables and fruits, olive oil, and moderate consumption of dairy products, fish, and poultry (3). It is associated with reduced risk of incident cardiovascular outcomes, including myocardial infarction, stroke, and atrial fibrillation; lower incidence of certain cancers, with improved survival; and improved metabolic endpoints, including type 2 diabetes, metabolic syndrome, and obesity (4). Mediterranean-style diets have been associated with reduced risk of neurocognitive conditions including depression, dementia and anxiety (5–7). A recent meta-analysis found that adherence to a Mediterranean diet was associated with reduced neurological and psychiatric endpoints including dementia, depression and anxiety (8). Similarly, another current meta-analysis demonstrated that better adherence to the EAT-Lancet diet (closely aligned with the Mediterranean diet) was associated with a 13% relative reduction in anxiety risk (9).

Mechanisms proposed to explain associations between Mediterranean-style diets and health outcomes include antioxidant potential, attributable to the increased intake of fruits, vegetables and olive oil (10); anti-inflammatory effects, driven primarily by olive oil, which has been shown to reduce markers of inflammation including C-reactive protein and pro-inflammatory cytokine interleukin-6 (11); and beneficial microbiome alterations related to the high fiber content of Mediterranean-style diets, especially increased levels of beneficial microbe populations and enhanced concentration of fecal short-chain fatty acids (12).

The association of Mediterranean-style diets and reduced prevalence of neurocognitive outcomes, particularly anxiety, is well established (13); however, whether the association persists when external circumstances are objectively stressful has been less well studied. A significant inverse association between the Mediterranean diet score (the degree to which the reported diet approximates the Mediterranean diet), measured using the Israel Mediterranean diet screener (I-MEDAS), and anxiety, measured using the General Anxiety Disorder-7 (GAD-7) screener, during the COVID lockdowns, indicating that closer adherence to a Mediterranean diet was associated with less anxiety (14).

Like any war, the Iron Swords war 2023–2025 between Gaza and Israel was characterized by tremendous uncertainty and social and psychological stress. Lifestyle patterns were disrupted, including alterations in diet. The purpose of the present study was to examine whether an association between diet quality, defined as consumption of a Mediterranean-style diet, and anxiety existed during a period of tremendous exogenous stress.

## 2 Materials and methods

### 2.1 Study design

The present cross-sectional study was conducted online in a representative sample of Israeli adults using structured surveys for data collection. Queried were diet quality, assessed using the Israel Mediterranean diet screener (I-MEDAS) (15); prevalent anxiety, measured using the General Anxiety Disorder (GAD)-7 (16) questionnaire; food insecurity, measured using the Hunger Vital Sign questionnaire (17); and demographic information.

### 2.2 Study setting and duration

The study was conducted online using a Google platform and managed by Sekernet, a provider of survey logistics and management services. Data were collected within shortly after the onset of the war, from November 6–14, 2023.

### 2.3 Ethical considerations

The study was approved by the Institutional Ethics Board of Ariel University, Israel (approval number AU-HEA-MB-20231105, November 5, 2023).

### 2.4 Study participants

Eligible for participation were all Israeli adults who indicated their age as 18 years or older, and who provided informed consent by clicking on the appropriate button. Excluded from participation were respondents who did not provide informed consent by clicking on the appropriate button, and those who indicated their age as younger than 18 years.

### 2.5 Recruitment or sampling method

The Israeli population was stratified by age, sex, and ethnicity. Random sampling was performed within each of the strata. Subjects thus identified were approached online by Sekernet, and a survey link was provided if the potential subject indicated interest in study participation. As stated, the subject could proceed with survey participation by clicking the button indicating informed consent; alternatively, the subject could decline participation. Survey recruitment and data collection were halted once > 500 responses were accrued. All data were de-identified.

### 2.6 Sample calculation

For each participant, the GAD-7 score was assigned to a category of anxiety as used in clinical practice: 0–4 = minimal anxiety; 5–9 = mild anxiety; 10–14 = moderate anxiety; 15–21 = severe anxiety. The categories were collapsed into two: 0–9 (minimal-mild anxiety vs. 10 or greater (moderate–severe anxiety). With a total sample size of 386 (*n* = 193 in each group) the present study had 80% power to detect a true, by-anxiety group difference in the total I-MEDAS score. The calculated sample size was increased by 30% to retain study power in the event of incomplete data, so the final sample size was *n* = 501. This calculation assumes a two-sided alpha of 0.05 and a total between group difference in I-MEDAS score 1 ± 3.5 points (based on prior experience). However, the survey company continued sampling to *n* = 534. This increases study power to 83%, with the same underlying assumptions.

### 2.7 Measures

#### 2.7.1 Anxiety: GAD-7

Self-reported anxiety was measured using the validated, Hebrew language translation of the General Anxiety Disorder (GAD)-7 survey (18). This 7-item questionnaire asks study participants to indicate the frequency with which each of the presented statements was true during the 2 weeks prior to the date of the survey. Each item on the scale can receive a score as follows: 0 (not at all); 1 (several days); 2 (more than half of the days); 3 (nearly every day); thus, the total score can receive a value from 0 to 21, where a higher score indicates greater anxiety. Cut-off scores for mild, moderate, and severe anxiety symptoms are 5, 10, and 15, respectively (18).

#### 2.7.2 Diet quality: I-MEDAS

The Israeli Mediterranean diet screener (I-MEDAS) is a 17-item questionnaire designed to assess the degree to which a respondent’s diet approximates Mediterranean diet principles (15). The screener is not intended to measure typical or specific intake; rather, it is intended to indicate diet quality. Thus, the higher the I-MEDAS score, the more similar the diet is to the Mediterranean diet and the better the diet quality. The items comprising the total I-MEDAS score are presented in Table 1.

**Table 1:** Adherence to individual I-MEDAS items by anxiety level.

I-MEDAS items	% receiving a point for the item	
	GAD-7 score < 10: minimal to mild anxiety (*n* = 322)	GAD-7 score ≥ 10: moderate to severe anxiety (*n* = 212)
Uses olive oil as main culinary fat	189 (73.6)	139 (65.6)
Eats poultry/white meat more than red meat	237 (73.3)	151 (71.2)
At least two vegetable servings/day	150 (47.8)	88 (42.3)
Three or more fruit servings/day	50 (15.5)	18 (8.5)
Less than one butter/margarine/cream servings/day	193 (59.9)	113 (53.3)
Less than one sugar-sweetened beverages/day	197 (61.2)	106 (50.0)
Three or more whole grain servings/day	59 (18.3)	51 (24.1)
Two or more unsweetened dairy servings/day	166 (52.5)	113 (53.6)
Fewer than seven red/processed meat servings/week	310 (96.3)	199 (93.9)
Seven or more alcoholic beverages/week	5 (1.6)	7 (3.3)
Three or more legume servings/week	88 (27.3)	58 (27.5)
Three or more fish servings/week	37 (11.5)	30 (14.2)
Three or more nut servings/week	95 (29.5)	60 (28.3)
Three or more hummus/tahina servings/week	122 (37.9)	76 (35.8)
Fewer than three sweet baked goods servings/week	142 (44.1)	84 (39.6)
Fewer than two savory baked goods servings/week	264 (82.0)	168 (79.2)
Three or fewer salty snacks servings/week	275 (85.4)	170 (80.2)

#### 2.7.3 Hunger vital signs

Food insecurity was measured using the Hunger Vital Sign two-item questionnaire (17). A participant who responded “sometimes true” or “often true” to either of the two questionnaire statements was categorized as food insecure (19). The two statements were:

- “Within the past 12 months, you worried whether your food would run out before you got the money to buy more,”
- “Within the past 12 months, the food you bought just did not last, and you did not have money to get more.”

#### 2.7.4 Demographic and lifestyle data

The following demographic data were collected: age, sex, highest level of education completed, ethnicity, religiosity, geographic location of residence, current smoking, minutes of exercise per week, change in diet compared to pre-war diet, change in body weight since war began, and difference in weight since war began.

### 2.8 Data collection procedures

Potential study participants were randomly selected from the general Israeli adult population using population lists stratified for age, sex, and ethnicity. Interested individuals were provided with a link to the study. Prior to initiating the survey, potential participants were asked if they were at least 18 years of age. If the response was negative, the survey terminated. Potential respondents indicating they were 18 years of age or older were directed to the informed consent statement, for which they provided consent by clicking on the appropriate button. At this point, the survey commenced.

### 2.9 Statistical analysis

Data were analyzed on SPSS Statistical Analysis Software v29. The Kolmogorov–Smirnov test was used to assess the normality of continuous variables. Normally distributed continuous variables were described as mean ± standard deviation, while variables with distributions significantly deviating from normal were described as median [interquartile range (IQR)]. Nominal variables were presented as *n* (%). The GAD-7 total score was calculated and categorized as equal to or greater than 10 (moderate to severe anxiety) vs. less than 10 (minimal or mild anxiety). Continuous variables were compared by GAD-7 category using the *t*-test for independent variables or the Mann–Whitney *U* as appropriate. Nominal variables were compared by GAD-7 category using the chi-square test. GAD-7 category was modeled using binary logistic regression analysis. GAD-7 was additionally analyzed as a continuous variable using linear regression analysis. All analyses were two-sided and considered significant at *p* < 0.05.

## 3 Results

The study population comprised 534 Israeli adults aged 18 years or older. Of these, 212 (39.7%) had GAD-7 scores of 10 or greater, defining them as moderately to severely anxious.

Table 2 displays characteristics of the study population by anxiety level. Participants with moderate to severe anxiety were almost a decade younger than those with minimal to mild anxiety. Approximately 45% more women than men had GAD-7 scores consistent with moderate to severe anxiety. Ethnicity differed significantly by anxiety level, driven by significantly greater than expected moderate to severe anxiety among Druz. A significantly smaller proportion of religious individuals (regardless of religion) had moderate to severe anxiety than secular or traditional respondents. Family status, the number of minor children living in the household, the location of the household, and the highest level of education completed did not differ by anxiety level.

**Table 2:** Study population demographic characteristics by level.

Characteristic	GAD-7 score < 10: minimal to mild anxiety (*n* = 322)	GAD-7 score ≥ 10: moderate to severe anxiety (*n* = 212)
Age [years, median (interquartile range)]	44 (27)	35 (19)
Sex, *n* (% female)	127 (39.4)	152 (71.7)
Family status, *n* (%)		
Married/domestic partnership	210 (65.2)	133 (62.7)
Single	79 (24.5)	62 (29.2)
Divorced	30 (9.3)	15 (7.1)
Widowed	3 (0.9)	2 (0.9)
Children in the household < 18 years of age [median (interquartile range)]	0 (2)	0.5 (2)
The region of your residence on October 7, 2023, *n* (%)		
Central region (excluding Tel Aviv)	73 (22.7)	51 (24.1)
Northern region	49 (15.2)	47 (22.2)
Tel Aviv	56 (17.4)	27 (12.7)
Southern region	38 (11.8)	25 (11.8)
Jerusalem	35 (10.9)	20 (9.4)
Haifa region	27 (8.4)	19 (9.0)
Judea and Samaria	20 (6.2)	11 (5.2)
Triangle (i.e., Jaljulia, Taibeh, Um al Fahem, Kfar Kara)	10 (3.1)	7 (3.3)
Other	14 (4.3)	5 (2.4)
Ethnicity, *n* (%)		
Jewish	269 (83.5)	164 (77.4)
Arab	43 (13.4)	37 (17.5)
Druze	4 (1.2)	10 (4.7)
Other	6 (1.9)	1 (0.5)
Religiosity, *n* (%)		
Secular	136 (42.2)	101 (47.6)
Traditional	97 (30.1)	76 (35.8)
Religious	89 (27.6)	35 (16.5)
Highest level of education completed, *n* (%)		
Fewer than 12 years	3 (0.9)	1 (0.5)
High school diploma/matriculation	31 (9.6)	16 (7.5)
Professional license (technician, tradesperson, etc.)	58 (18.0)	47 (22.2)
Bachelor’s degree	87 (27.0)	56 (26.4)
Master’s degree or higher	82 (25.5)	59 (27.8)
Other	61 (18.9)	33 (15.6)

Table 3 presents lifestyle characteristics of the study population by anxiety level. A significant difference in weekly minutes spent exercising, though it is difficult to perceive using the median (IQR). To more easily intuit the difference, the mean in the minimal to mild anxiety group was 115.26 ± 133.33, compared to 96.74 ± 124.79 in the moderate to severe group. Weekly minutes of physical activity were significantly lower than before the war (*p* < 0.001) and significantly lower in the moderate-to-severe group than in the minimal-to-mild anxiety group. There were 37% more current smokers in the moderate to severe anxiety group than in the minimal to mild group. A significantly greater proportion of individuals in the moderate to severe anxiety group reported weight change, both weight gain and weight loss, than less anxious respondents. On the other hand, the amount of weight gained or lost did not differ between the two groups. A by-anxiety level difference in the I-MEDAS score was not detected; however, a significantly greater proportion of people in the moderate to severe anxiety group reported a deterioration of diet quality since the onset of the war, characterized by less structure, more snacking, and eating fewer servings per day of fruits and vegetables. Additionally, a significantly greater proportion of participants in the moderate to severe anxiety group reported food insecurity by responding “often true” or “sometimes true” to one or both of the statements. A total of 44 (8.2%) participants experienced food insecurity using the Hunger Vital Sign definition. The proportion of respondents reporting food insecurity in the moderate to severe anxiety group was more than twice that of the minimal to mild anxiety group.

**Table 3:** Lifestyle and dietary characteristics of the study population by anxiety level.

Characteristic	GAD-7 score < 10: minimal to mild anxiety (*n* = 322)	GAD-7 score ≥ 10: moderate to severe anxiety (*n* = 212)
Minutes of exercise/week prior to war [median (IQR)]	60 (143)	60 (110)
Minutes of exercise/week in the past week [median (IQR)]	60 (140)	20 (90)
Present smoker (at least one cigarette smoked per day) *n* (%)	74 (23.0)	67 (31.6)
Weight change since the start of war *n* (%)		
No weight change	150 (46.6)	44 (20.8)
Yes, weight gain	87 (27.0)	93 (43.9)
Yes, weight loss	30 (9.3)	39 (18.4)
Do not know	55 (17.1)	36 (17.1)
Quantity of weight change among those reporting change (in kg)		
Weight gained [median (IQR)]	2 (1)	2 (1)
Weight lost [median (IQR)]	−2.5 (2.1)	−3 (1.4)
Change in diet quality *n* (%)		
No difference	191 (59.3)	82 (38.7)
Worse quality since the war began	55 (17.1)	62 (29.2)
Healthier now during the war	15 (4.7)	12 (5.7)
Less structure now	55 (17.1)	62 (29.2)
More snacks now	44 (13.7)	61 (28.8)
Fewer fruits/vegetables now	26 (8.1)	38 (17.9)
I-MEDAS score [median (IQR)]	8 (4)	8 (3)
Food insecure, *n* (%)*	16 (5.0)	28 (13.2)

I-MEDAS, Israel Mediterranean diet screener; IQR, interquartile range.

^*^Food insecure defined as responding yes to either or both of the Hunger vital sign questions.

Adherence to each item comprising the I-MEDAS score is shown by anxiety level in Table 1. Overall, study population compliance is strongest for consuming fewer than two savory baked goods each week and three or fewer salty snacks each week. A stated preference for olive oil rather than other fats and poultry rather than red meat was also reported by a large proportion of participants. The least adhered to item was consuming seven or more alcoholic beverages per week. Poor adherence was also observed for consuming three or more servings each of fruit, whole grains, and fish. Despite a lack of difference in total I-MEDAS score, differences in the proportion of participants adhering to some of the items were observed; specifically, a significantly smaller proportion of participants consumed the prescribed number of fruit servings in the moderate to severe anxiety group compared to the minimal to mild anxiety group. Similarly, a significantly smaller proportion of participants in the moderate to severe anxiety group reported consuming less than one serving a day of sugar-sweetened beverages than less anxious participants.

Moderate to severe anxiety was modeled using binary logistic regression analysis, as shown in Table 4. Each 1-year increase in age was associated with a 2% relative reduction in odds of moderate to severe anxiety. The odds of moderate to severe anxiety were 3.875 times greater in women than in men. Individuals who reported that their current diets had deteriorated in quality since the beginning of the war had a relative increase of 68% in odds of moderate to severe anxiety. Also predictive of moderate to severe anxiety was weight gain, such that people who reported gaining weight had a relative increase of 83% in the odds of this outcome. Food insecurity, measured by the Hunger Vital Sign, increased the odds of moderate to severe anxiety by a factor of almost 2.5. By contrast, religiousness was protective, reducing the odds of moderate to severe anxiety by a relative 50.8%. The model was significant (*p* < 0.001) and correctly classified 69% of study participants for anxiety level.

**Table 4:** Binary logistic regression of moderate to severe anxiety (GAD-7 score ≥ 10).

Variable	Odds ratio	95% confidence interval	*p*-value
Age (years)	0.980	0.968–0.993	0.003
Female sex	3.875	2.605–5.763	<0.001
Religious	0.492	0.304–0.796	0.004
Diet quality worse since start of war	1.684	1.055–2.689	0.029
Food insecure	2.489	1.230–5.037	0.027
Weight gain since start of war	1.831	1.214–2.762	0.004
Constant	−1.776		0.013

The model was significant (*p* < 0.001) and correctly classified 69% of participants for moderate to severe anxiety.

The same variables were included in a linear regression of the GAD-7 score as a continuous variables, and the following equation was obtained: GAD-7 = 13.5–0.07 (age) – 6.63 (sex where male sex = 1, female sex = 0) + 2.75 (weight gain, 1 = yes, 0 = no) – 7.61 (religiosity where a higher score indicates greater religiosity) + 3.41 (food insecurity, where food insecurity = 1 and no food insecurity = 0). The variables weight gain and worsened diet quality since the onset of the war were not significantly included in the equation. The regression itself was significant (*p* < 0.001) and explained approximately 25% of the variability in the GAD-7 score.

## 4 Discussion

The present study was designed to assess whether an association exists between diet quality, defined as consumption of a Mediterranean-style diet, and anxiety, measured using the GAD-7, during a period of armed conflict. A direct association was not detected insofar as the total I-MEDAS score did not significantly differ by anxiety level. On the other hand, two specific items in the I-MEDAS did differ by anxiety level: a significantly greater proportion of people with moderate to severe anxiety reported not consuming three or more servings of fruit daily and did not drink less than one sugary beverage daily.

Though the total I-MEDAS score did not differ, other measures of diet quality did differ by anxiety level: reporting that diet quality has deteriorated since the war began, a less structured diet with more snacks and fewer servings of fruits and vegetables, and a change in weight (either gain or loss). In the logistic regression analysis, age and religiosity reduced odds of high levels of anxiety while female sex, worsening of diet quality, food insecurity and weight gain all increased odds of moderate to severe anxiety. A reduction of specific expressions of anxiety with increasing age has been reported in meta-analyses. In the same meta-analysis, the persistence of greater anxiety in women regardless of age was also observed (20). The emergence of emotional eating as a facilitator of weight gain supports a role for reduced diet quality and weight gain in anxiety (21). However, the direction of this association is unclear. It is possible that anxious individuals consume more food to reduce anxiety, but it is also possible that consuming a poor quality diet and weight gain increase anxiety risk. The association between food insecurity and anxiety has been reported (22); however, here, too, the direction of the association remains unclear. Certainly, food insecurity can increase anxiety; but it is also possible that anxious individuals are more likely to identify as food insecure. The measure of food insecurity in this and many other studies is self-reported and may be more likely to be reported as existing in particularly anxious persons. The association between religiosity and anxiety has also been documented. In a meta-analysis of religion and spirituality in the treatment of depression and anxiety, treatments including religious and spiritual elements were found to be effective (23). Findings of the logistic regression were echoed in the linear regression of the continuous GAD-7 score; however, in this analysis, worsening of diet quality since the onset of the war was not included in the analysis, which may reflect a small or inconsistent slope across the distribution of the continuous GAD-7 variable. Still, associations between anxiety and diet quality have been observed in several populations. For example, anxiety was associated with more disordered eating and poorer diet quality in young athletes (24).

A disruption of healthier eating patterns has been reported in populations undergoing armed conflict, including the Iron Swords War. Individuals attending a weight management clinic reported a change in body weight approximately 3 months after the war began. Compared to those who did not experience weight gain, those with an acute weight gain of > 3% reported disrupted sleep, increased anxiety and psychological distress, anxiety-driven eating, including more snacks and sweets, and less physical exercise (25).

Inverse associations between adherence to the Mediterranean diet and anxiety have been reported in a number of populations. For example, a Mediterranean-style diet was shown to reduce symptoms of depression and anxiety in healthy Iranian adults working for government agencies (26); obese adults attending the International Center for the Assessment of Nutritional Status at the University of Milan (27); Chinese adults aged 65 years or older (28); Turkish adults (29); and others, though a number of neuroprotective mechanisms, including enhance microbial diversity, decrease oxidative stress and inflammation, and improved communication along the gut-brain axis (30).

In addition to observational studies, clinical trials have demonstrated the efficacy of Mediterranean diets in anxiety reduction. A Mediterranean-style diet intervention initiated at the midpoint of a high-risk pregnancy significantly reduced anxiety by the last month of the pregnancy in pregnant women compared with usual care (31). A clinical trial conducted in adults with a history of psychological stress and anxiety found a significant reduction in anxiety among individuals randomized to a Mediterranean diet compared to those who received a standard control diet (32). Compared to a control calorie-deficient diet, a low-calorie Mediterranean diet reduced anxiety in reproductive-aged women with polycystic ovarian syndrome (33).

### 4.1 Limitations

Findings of the present study must be considered in the framework of methodological limitations. First, the study population, while randomly selected from relevant strata and thus representative of the general Israeli population, nevertheless reflects volunteer bias. It is possible that interest in nutrition, health, and/or anxiety or some other characteristic motivated some individuals to agree to participate. This would negatively influence the external validity of the study. Also, the study was conducted online, precluding the participation of adults with poor digital literacy or lacking access to a computer or smartphone. On the other hand, Israel has > 100% penetration of cell phone use, among the highest in the Organization for Economic Cooperation and Development (OECD).^1^ Similarly, mobile internet penetration is currently greater than 95%,^2^ suggesting that despite these possible sources of bias, the study population was nevertheless likely representative of its target.

Another limitation is the cross-sectional study design. Without knowledge of changes in diet quality from prior to the onset of the war, it is not possible to know whether diets became less aligned with the Mediterranean diet framework. This may explain why a correlation between anxiety and the I-MEDAS score was not detected. However, strong associations between measures of diet deterioration and anxiety were observed, including a stated worsening of diet quality; less dietary structure; eating fewer daily servings of fruits and vegetables; and consuming more snacks.

## 5 Conclusion

A deterioration of diet quality, but not the I-MEDAS score, was associated with moderate to severe anxiety in a representative sample is Israeli adults during a period of armed conflict. Due to the cross-sectional study design, it is not possible to assign causality; specifically, it is not clear whether consuming a poorer diet leads to greater anxiety, whether greater anxiety leads to deteriorating diet quality, or whether a third variable explains variability in both diet quality and anxiety. Regardless of causality, biological mechanisms exist to explain the association. Also independent of causality is public health messaging, that could target both diet quality and anxiety during periods of war.

## Data Availability

The raw data supporting the conclusions of this article will be made available by the authors, without undue reservation.

## Footnotes

1
https://worlddata.info/asia/israel/telecommunication.php

2
https://www.statista.com/statistics/974320/israel-mobile-phone-internet-user-penetration/
